# A cluster randomized controlled trial of extending ART refill intervals to six-monthly for anti-retroviral adherence clubs

**DOI:** 10.1186/s12879-019-4287-6

**Published:** 2019-07-30

**Authors:** Lynne Wilkinson, Anna Grimsrud, Tali Cassidy, Catherine Orrell, Jacqueline Voget, Helen Hayes, Claire Keene, Sarah Jane Steele, Rodd Gerstenhaber

**Affiliations:** 1 Center for Infectious Disease and Epidemiological Research, School of Public Health and Family Medicine, University of Cape Town, Cape Town, South Africa; 2 International AIDS Society, Cape Town, South Africa; 3 Medécins Sans Frontières, Cape Town, South Africa; 4 Division of Public Health Medicine, School of Public Health and Family Medicine, University of Cape Town, Isivivana Centre, 8 Mzala Street, Khayelitsha, Cape Town, South Africa; 5 Department of Medicine, Faculty of Health Sciences, Cape Town, South Africa; 6 The Desmond Tutu HIV Centre, Institute for Infectious Disease and Molecular Medicine, University of Cape Town, Cape Town, South Africa; 7 Western Cape Government Department of Health, Cape Town, South Africa

**Keywords:** Extended dispensing intervals, Antiretrovirals, Differentiated service delivery, HIV

## Abstract

**Background:**

The antiretroviral therapy (ART) adherence club (AC) differentiated service delivery model, where clinically stable ART patients receive their ART refills and psychosocial support in groups has supported clinically stable patients’ retention and viral suppression. Patients and health systems could benefit further by reducing visit frequency and increasing ART refills. We designed a cluster-randomized control trial comparing standard of care (SoC) ACs and six-month ART refill (Intervention) ACs in a large primary care facility in Khayelitsha, South Africa.

**Methods:**

Existing ACs were randomized to either the control (SOC ACs) or intervention (Intervention ACs) arm. SoC ACs meet five times annually, receiving two-month ART refills with a four-month ART refill over year-end. Blood is drawn at the AC visit ahead of the clinical assessment visit. Intervention ACs meet twice annually receiving six-month ART refills, with a third individual visit for routine blood collection anytime two-four weeks before the annual clinical assessment AC visit. Primary outcomes will be retention in care, annual viral load assessment completion and viral load suppression. (<400copies/mL) after 2 years.

Ethics approval has been granted by the University of Cape Town (HREC 652/2016) and the Medecins Sans Frontieres (MSF) Ethics Review Board (#1639). Results will be published in peer-reviewed journals and made widely available through presentations and briefing documents.

**Discussion:**

Evaluation of an extended ART refill interval in adherence clubs will provide evidence towards novel model adaptions that can be made to further improve convenience for patients and leverage health system efficiencies.

**Trial registration:**

Registered with the Pan African Clinical Trial Registry: PACTR201810631281009. Registered 11 September 2018.

**Electronic supplementary material:**

The online version of this article (10.1186/s12879-019-4287-6) contains supplementary material, which is available to authorized users.

## Background

South Africa is home to the largest number of people living with HIV (PLHIV), an estimated 7.9 million people. In 2017, it was estimated that 55.7% of PLHIV in South Africa were on antiretroviral therapy (ART) [1]. In February 2018, the South African president committed to starting another 2 million PLHIV by 2020 towards ensuring South Africa meets its target of 81% by the end of 2020 [2, 3]. To achieve these targets, the already overburdened health system needs to find ways to attract and retain significantly more patients on ART.

Differentiated models of ART delivery for patients that are otherwise healthy and clinically stable on ART, attempt to make ongoing access to ART refills and clinical management more convenient and easily accessible in order to support continued adherence to treatment on a long-term basis. Such models have been shown to be feasible to implement, acceptable to patients with good retention and viral suppression outcomes. These models can also be leveraged to improve health system efficiency in an era with limited resources to achieve ambitious targets [4–8]. One such differentiated ART delivery model is the ART adherence club (AC), originally a demonstration project by Médecins Sans Frontières (MSF) in Khayelitsha, South Africa. The AC model was created to and has supported long-term adherence and retention of clinically stable ART patients [4, 9–14]. It has been endorsed in a number of sub-Saharan African country policies [15–17], including South Africa, where it has been implemented at scale [18].

Novel adaptations to differentiated ART delivery models such as ACs are needed in order to further increase convenience and access for patients and expand ART access within a context of declining resources [19]. One possible adaptation is to reduce the frequency of visits by increasing the amount of ART dispensed at each refill. This would allow for an increase in the number of ACs managed by the same staff complement, and could further decongest facilities, making space for new patients to start ART and for patients receiving an ART regimen that is failing and those presenting to care with advanced HIV disease to be more intensively clinically managed.

In the World Health Organisation’s (WHO) updated 2016 consolidated guidelines on the use of antiretroviral drugs for treating and preventing HIV infection, WHO recommends that clinically stable patients can receive ART refills every 3–6 months [20]. However, there is a paucity of retention and viral suppression evidence supporting longer intervals (e.g. 4–6 months). Evidence beyond 3-month ART refills is needed before health authorities invest in changing their supply chain management system to support such longer ART refills periods (see concerns raised in pre-study engagment, Additional file 1). This study aims to provide high-quality evidence on the effect of extended ART dispensing intervals on sustained retention in care and viral suppression.

## Methods

### Study aims & objectives

The overall aim of this project is to investigate the impact of extended ART dispensing intervals and less frequent psychosocial support on retention in care and viral suppression among clinically stable ART patients in ACs.

1. Primary objective
  To compare the retention, viral load assessment completion and viral suppression outcomes of patients receiving their ART refill through standard of care (SOC) ACs and six-month ART refill (Intervention) ACs over 24 months.
2. Secondary objectives

To determine:

- Medicine supply chain feasibility of Intervention ACs implementation, including medicine losses and wastage associated with the six-month supply of ART.
- Impact of Intervention ACs model on clinic congestion.

### Study design

This is a cluster-randomized control trial to evaluate whether patients in Intervention ACs have non-inferior retention and viral load assessment and viral suppression outcomes compared to those in SOC ACs.

### Setting

The study will take place in Khayelitsha, a peri-urban area home to approximately 500 000 people. The community is of low socioeconomic status with high levels of HIV (antenatal HIV prevalence of 34% in 2016) and high levels of unemployment [21]. The participants will be recruited from Ubuntu Site B clinic’s existing ACs. A summary of the study site is provided in Table 1.

**Table 1:** Summary of study site

Site	Total on ART at site end March 2017	Date ACs started at site	Total adults in ART ACs end March 2017
Ubuntu ART clinic, Khayelitsha	10252	2007	4535

### Study population

Participants will be stable HIV-positive adults on ART who are currently in an AC at the study site. ACs will be recruited at their routine AC visit, which occurs five times per year. Study inclusion criteria exist at both AC and individual level.

#### AC inclusion criteria

- > 90% of patients in the AC consent to be enrolled in study prior to randomization

#### AC exclusion criteria

- ACs catering to patients who require more regular clinical follow-up than a yearly visit (e.g. family ACs, youth ACs)
- ACs facilitated by nurses with limited group interaction/ support and/or which function more as ART pick-up point (e.g. evening ACs)

#### Participant inclusion criteria

- Age 18 years or older
- On ART for at least 6 months
- Most recent documented viral load < 400 copies/mL (not older than 12 months)
- Able to provide informed consent for research

#### Participant exclusion criteria

- Intention to relocate out of Cape Town permanently during the study period

### Description of intervention and control

A description of SOC ACs and Intervention ACs is provided below and summarized in Table 1.

#### The SOC ACs

Participants in the SoC ACs will continue to be provided with their ART refill, clinical care and support following the current Western Cape provincial AC guidelines. At time of protocol submission, SOC ACs were groups of 25–30 stable patients who had been on ART for 6 months or more, had an undetectable viral load, and were referred by a clinician to the model of care. SOC ACs are facilitated by a lay healthcare worker and meet five times per year (bi-monthly with a four-month gap over the end of the year to support migration patterns) for a group support session, a brief symptom check and distribution of pre-packed ART with referral by the lay provider to a nurse based at the clinic if necessary. At one of these visits the patient’s blood is taken by a nurse for routine blood monitoring, including viral load. At the following AC visit, each AC member has their annual clinical assessment with a nurse.

Patients are allowed to send a treatment “buddy” to collect their ART refill to every alternate AC. A treatment buddy is a friend or family member of an AC member requested by the AC member to collect their ART refill at from the AC on the AC member’s behalf.

#### The intervention ACs

The Intervention ACs meet twice annually receiving six-month ART refills, with the patient being asked to individually attend a third visit for routine blood collection anytime two-four weeks before their annual clinical assessment AC visit. Patients in Intervention ACs are not able to send a treatment “buddy” to collect their ART refill to ensure the health system interacts with patient at least twice a year.

In both SOC and Intervention ACs, a five-day grace period is permitted for late attendance. In other words, AC patients need to come within 1 week of their AC visit to collect their ART refill or they are up-referred to the clinician-led ART care based at the clinic. A patient is also up-referred if they experience viral rebound (a viral load of > 400 copies/mL) or develop another clinical condition that requires more regular clinical support (Table 2).

**Table 2:** Comparison of SOC ACs and Intervention ACs

	Standard of Care ACs	Intervention six-month ACs
Frequency of AC visits	2-monthly (5 per year)	6-monthly (2 per year)
ART dispensing interval	2-monthly (5 per year)	6-monthly (2 per year)
Frequency of clinical assessments	12-monthly	12-monthly
Frequency of routine bloods	12-monthly	12-monthly
Timing of routine bloods	As part of AC visit	As an additional individual visit, 2–4 weeks before clinical assessment AC visit
Units of care	Groups of 25–30	Groups of 25–30
Peer-based support	Strong emphasis	Strong emphasis
Patient self-management	Strong emphasis	Strong emphasis
Management of clinical complications	Up-referral to clinician-led ART care based at the clinic	Up-referral to clinician-led ART care based at the clinic
ART packing and dispensing	Pre-packed by central dispensing unit, supplied to clinic pharmacy and dispensed at AC visit	Pre-packed at clinic pharmacy with support from study team staff and dispensed at AC visit
Treatment “buddies”*	Allowed to collect at every alternate AC visit	Not permitted
Standard number of contacts per year	5 (all within the AC)	3 (2 within the AC and 1 individual for routine bloods)
Minimum number of contacts per year	3 (could send a “treatment buddy” to collect ART twice)	3 (within the AC and 1 for routine bloods)

### Recruitment - study participation and withdrawal processes

ACs eligible for the study will be offered study participation at a routine AC visit. After the study is explained, the group will be afforded an opportunity to discuss and ask questions. Thereafter each AC member present will be asked to cast their individual vote for study participation on a named voting slip, which will folded and placed in a closed box handled by the study staff. The study staff will exit the venue to count the votes and will return to inform the group of the final outcome (i.e. > 90% voted to participate or ≤ 90% voted to participate) with each individual’s vote remaining confidential and only known to study staff who will not disclose it to any other person.

If more than 90% of the AC members vote in favour of participating in the study, the AC will be enrolled in the study. Consent procedures will take place at the same AC visit, or over the subsequent one or two visits, if patients need more time to consider or were not present at the study enrolment AC visit.

In the case where an individual patient does not vote in favour of study participation, the patient will have the choice to stay in the AC and participate in the study or to transfer to a newly formed AC that is not participating in the study.

AC randomization will take place after all AC patients have provided consent, or arranged to transfer out. Randomization will be performed with the *Randomize* package in Stata, ensuring balance between community and facility ACs in each arm [22]. At the start of the following AC meeting, the study staff will draw an envelope containing the randomization outcome and inform the AC members whether their AC has been allocated to the SOC or intervention arm (see randomization SOP in Additional file 3). The ACs will vote on their continuation in the study for a second time, following the same procedure as the first vote.

At the AC visit after being informed of their AC study arm, groups will be given their first study ART refill.

**Fig. 1:**
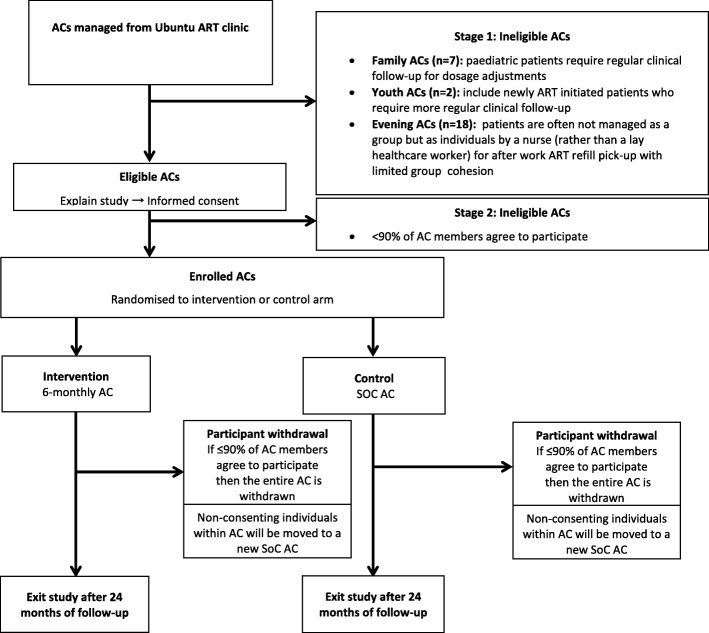
Study Schema

See Fig. 1 for more details.

### Sample size and power considerations

As this study is interested in non-inferiority, all statistical tests were one-tailed with an alpha of 0.025 to address type 1 error. At time of protocol development, there were 97 eligible ACs. We assumed 10% of the ACs were would not participate in the study leaving 87 ACs eligible for randomization (approximately 43 per arm), with a total of 1986 participants (993 per arm). We proposed a clustered sampling strategy for our study. There is a paucity of information in the literature regarding correlation between individuals within ACs, therefore we reviewed the literature relating to the clustering effect of facilities. Thus, to account for the correlation within clusters we assumed a correlation ranging from 0.05–0.10 and an average cluster size of 24 [23, 24] resulting in our estimated design effect (deff) ranging from 2.15–3.3. We applied the deff to our total sample size to determine the effective sample size. Assuming retention in care would range from 75 to 85% with a 10% difference in retention in care between the control and intervention groups, we estimated the power of our study to range from 0.80–0.98. See supplementary materials for detailed sample size calculations.

### Primary outcome measures

Primary outcomes are retention in Ubuntu clinic care (AC or clinic care), retention in AC care, annual viral load completion rates, and VL suppression (VL < 400 copies/ml).

### Secondary outcome measures

#### Medicine supply chain feasibility measures

1. Patient related:
  - number of study participants whose regimen was switched during the study (including date of switch);
  - number of study participants reporting ART medicine losses and the reported reason for such losses including, but not limited to, theft, damage or destruction in the home or unauthorized loan or provision to another person who ran out of ART;
  - number of study participants requests for additional ART refill supply from the clinic pharmacy in between AC visits and the reasons for such requests.
2. System related:
  - number of short supplies due to medicine supply shortages, supply chain gaps and expiry date related losses;
  - medicines quantities wasted due to any of: expiry, re-issue, regimen switch, non-collection or any other reason.

#### Clinic patient congestion impact measures

Number of clinic visits per annum (AC and non-AC visits) for intervention arm and SOC Arm.

### Data collection

Following recruitment, ACs that are eligible, consent, and are randomized to the intervention arm will be transitioned to Intervention ACs. Duration of follow-up for patients is identical for patients in both arms of the studies.

Primary outcome data will be extracted from routine clinical records including AC registers, the National Health Laboratory Services, and patient paper and electronic records. Data will be collected for 27 months: 24 from the start of the study, with three additional months of follow-up before closing the dataset to ascertain final outcomes. This will be supplemented by data from the Western Cape provincial health data center, which uses probabilistic matching algorithms to link patient data from different Western Cape facilities, laboratory systems and pharmacy data.

Secondary outcome data related to medicine supply chain feasibility will be collected by the study team in collaboration with the pharmacy staff at the study site onto a lost drug reporting form, and will be compiled in a study database.

Additional data on ART dispensing between study visits and secondary outcome data related to patient visit data will be extracted from the provincial health data system.

Collection of participant names and other identifiers will be restricted to informed consent documents, and a study identification key, all of which will be kept in a locked cabinet in the study office at MSF separate from other study documentation and accessible only by the MSF project coordinator and PI. These records will be destroyed 3 years after completion of the final study report by a company that destroys confidential patient documents. All electronic records will be kept in password-protected files. All electronic communications of study data will be through password-protected, encrypted files.

### Data analysis

#### Primary outcomes

Analysis will be completed using STATA 14 [22] and will include: descriptive summary statistics of baseline patient characteristics, Kaplan-Meier estimates of time to loss to follow-up and first unsuppressed viral load, and calculation of the risk and hazards ratios at 12 and 24 months.

“Retention in care” will be defined as the proportion of patients retained in ART care at Ubuntu Clinic, in or out of an AC. If a patient transferred out of Ubuntu clinic before 24 months, he/she will be excluded from the denominator. “Retention in club care” will be defined as remaining a member of the original study AC at 24 months. If a patient leaves transferred out of their AC before 24 months, he/she is excluded from the denominator.

“Lost to follow up” is defined as patients failing to return to clinic or AC care for 3 months after a missed visit. “Viral load completion” is defined as having a viral load drawn within the first and second 12 month period after study start. If a patient is lost to follow-up, transferred out of clinic, or dies they are excluded from the denominator. “Viral load suppression” is defined as < 400 copies/mL. Only those patients with a completed viral load and retained in care at 24 months, are included in the denominator for viral load suppression. Patients who die or who are transferred out will be censored at the date of this outcome.

### Interim analyses

12-month primary outcomes will be assessed. An independent Data Monitoring Committee will conduct an interim review of the study patient primary outcomes. Should there be evidence of inferiority, it will be in a position to recommend stopping the study early (and disseminating study findings rapidly).

#### Secondary outcomes

Impact on intervention on clinic congestion will be evaluated by determining the total number of clinic visits per annum (AC and non-AC visits) by intervention arm participants compared with SOC arm participants. This data will be used to calculate an inferred annual deferred headcount.

## Discussion

### Benefits

There are both direct and indirect economic benefits to patients for participating in the study. If allocated to the intervention arm, by extending the interval between AC meetings, the financial burdens of transport, childcare costs and possible lost employment on patients is reduced. The study’s results could have further indirect benefit by stimulating policy changes, which would extend these benefits to all study participants in the long term and possibly other ART patients in the province and country.

### Risks

#### Undue group influence risk

Carrying out an intervention study in a group focused model of care requires a careful balancing of both the group and individual rights to participate or not participate in the study. By recognising that the group itself has a right to benefit from study participation and not to dissolve as result of study participation or non-participation, introduces a risk of undue influence over the individual to consent or refuse study participation. As outlined above in the methods section, the study design and procedures will limit this risk as follows:

- High thresholds (> 90%) have been set requiring an overwhelming majority of the group to be in favour of study participation for the AC to be included in the study.
- An additional step with similar thresholds was put in place for AC members to have a further opportunity to consider the AC’s study participation after being informed of the outcome of randomization.
- When the study is introduced to the group, the study staff will explain that all AC members have the right to refuse participation and doing so will in no way compromise their access to HIV management services.
- Voting will be confidential.

#### Adherence risk

While the evidence set out above demonstrates that there is limited risk that adherence will significantly decline due to less frequent engagement with the health system and with HIV positive peers on ART, this remains a risk. The risk will be minimized by adherence counseling at the AC meetings that focuses on remaining adherent during the long gap between meetings. Additionally, patients will be sent an SMS reminder of their AC appointment date 2 weeks before the appointment date. Patients who do not attend within the AC grace period in both arms will be contacted to remind them of their missed appointment.. Viral loads will be monitored annually and patients who are non-adherent and have a non-suppressed viral load will be up-referred back to clinician-led ART care at the main clinic for enhanced adherence and clinical support. Patients will also be encouraged and supported to make contact or meet up with other AC members outside of routinely scheduled AC meetings if this may be regarded to strengthen or maintain their support system.

#### Medicine loss risk

MSF has been running ACs at community venues from 2010 and from patient homes from 2012 without any reported incidents of theft. In addition, AC members have collected their ART refills from such community venues or other AC member’s homes and returned to their own homes without any reported incidents of theft. AC members have stored up to 4 months of ART received at year end in their informal housing (including shacks) in informal settlements since this provision of four-month ART refills over year end in 2012 with no increased reporting of medicine losses due to vulnerable living circumstances. For these reasons, there appears to be no increased risk of losses of medicine due to theft or damage or destruction within informal housing settlements.

Study staff will also assure study participants that there will be no negative outcome should any theft, damage, destruction or other loss take place and inform the participating AC members that should this occur they should immediately report such losses to their AC facilitator and/or any staff member at the Ubuntu clinic and/or any study staff and their lost ART medicines will immediately be replaced.

#### Medicine stability risk

Both the Western Cape Department of Health and MSF procure South African registered antiretroviral medicines (ARVs) that meet the requirements for long-term and accelerated testing as set out in the South African stability guidelines^1^ which requires that they are proven stable at 25 °C/60 RH for the entire shelf life and for 6 months at 40 °C /75 °C without loss in efficacy. Khayelitsha’s average maximum temperatures do not exceed 29 °C in February, which allows scope for peak temperatures and increased temperatures inside informal settlement environments. The risk will be minimized by educating all study participants to store their ART supply away from direct sunlight or source of heat and dry place within their home.

## Trial Status & Dissemination

### Timelines

This manuscript is an abridged version of Version 1 of the protocol, which was finalized in December 2016. We are currently still following patients up, and the final 24-month analysis will begin once data collection in complete in early 2020. Key dates are outlined below and in the SPIRIT figure in Table 1 (see full SPIRIT checklist in Additional file 4, Table 3).

- Submission to Ethics Committee and Ethics Approval: Aug-Nov 2016
- Presentation of study and AC study enrolment at AC meetings: Feb-June 2017
- Randomization outcomes and AC withdrawal process at AC meetings: Jul/Aug 2017
- First 6-month supply: Oct/Nov 2017
- Second 6-month supply: Mar/April 2018
- Interim data analysis March 2019
- Data Monitoring Committee review March 2019
- Data collection: Oct 2017-Sept 2019
- Data analysis: Oct - Dec 2019
- Submission for publication: March 2020

**Table 3:**
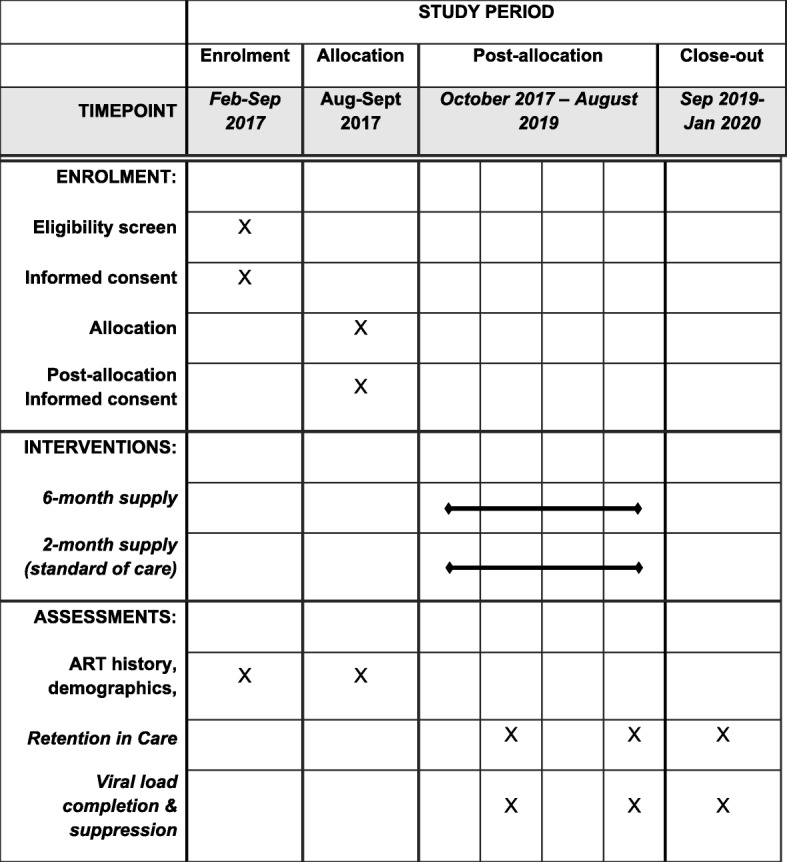
SPIRIT Figure of study procedure timelines

### Dissemination

The results of this study will be submitted to a peer-reviewed journal for publication and to local and international conferences. Results will also be disseminated to Western Cape Department of Health, the City of Cape Town, and other national ministry of health staff, patient advocacy groups, and at appropriate HIV/AIDS related conferences or forums.

## Additional files

Additional file 1: Pre-study engagement processes. Description of the various engagements and consultations that took place before study protocol was submitted (DOCX 15 kb)

Additional file 2: Patient Informed Consent form. Consent form used for study (DOCX 37 kb)

Additional file 3: Randomization SOP. The Standard operating Procedure for how randomization was performed during study. (DOC 132 kb)

Additional file 4: SPIRIT Checklist. (DOC 119 kb)

## Data Availability

All patient data used in this study is routinely captured by clerks at facilities. Researchers can access this data, with all linked pharmacy and laboratory results, from the Western Cape’s provincial health data center, by applying to the provincial research committee.

## Abbreviations

AC: Adherence club
ART: Antiretroviral therapy
Deff: Estimated design effect
MSF: Medecins Sans Frontieres
PLHIV: People living with HIV
SOC: Standard of care
WHO: World Health Organisation’s

## Footnotes

1 South African stability guidelines issued by the Medicines Control Council version 7.1
