# Supplementary material for: A cluster randomized controlled trial of extending ART refill intervals to six-monthly for anti-retroviral adherence clubs
Source: BMC Infect Dis. 2019 Jul 30;19:674. doi: 10.1186/s12879-019-4287-6 (PMC6664572; doi:10.1186/s12879-019-4287-6)
Supplement: Supplementary file 1 — Pre-study engagement processes. Description of the various engagements and consultations that took place before study protocol was submitted (DOCX 15 kb) [file 12879_2019_4287_MOESM1_ESM.docx]

# Appendix A – Pre-study engagement processes

**AC patients**

MSF piloted extending 2-month ART refills to 4-month ART refills over year end in 2012. This practice was adopted as policy in the Western Cape in 2013. AC patients are generally regarded to find this practice highly acceptable and throughout the Cape Metro district repeatedly enquire why this longer refill only takes place over year end and not throughout the year [18].

For this reason, MSF club facilitators attended a number of AC meetings both at Ubuntu ART clinic and in community and home venues to discuss acceptability of extending ART refills to 6 months with broad acceptability indicated in such discussions.

**Department of Health**

***Facility level***

A number of meetings have been held in 2016 with the Ubuntu ART site facility manager, club manager, pharmacist and other clinical staff to discuss the acceptability and buy-in for possibly providing 6-months of ART to stable patients if retention and viral suppression outcomes are not negatively affected by such a change in practice. The facility team has provided their buy-in and support.

***District level***

A number of meetings were held in 2015 with Khayelitsha district team, specifically the district manager, programmes co-ordintor and HAST medical officer. While the district manager initially expressed concern with patients’ capacity to manage longer ART refill supplies, after considering the existing evidence, the study design and discussing extensively with his district and facility team, the district manager indicated support for the study to the provincial department and in fact took up a position advocating for the provincial department to approve the study going ahead.

***Provincial level***

At provincial level there have been a number of engagements with different departments (HAST and pharmaceutical services), operational primary healthcare management and programmes management over a period of 12 months from mid-2015 to mid-2016.

While both the HAST and programmes departments largely supported the study going ahead, there was initially extensive caution and resistance from pharmaceutical services and operational primary health care management. These concerns centred around implementation expectations should study outcomes be positive and consequent changes required to drug supply chain management and budgetary cycles.

After considerable presentation of existing evidence, WHO 2016 recommendations and study design, the study was supported provided a senior pharmacist from pharmaceutical services and HAST department were included as co-investigators to provide input into study design to ensure inclusion of supply-chain feasibility related outcomes (secondary objectives) and agreement to constitute a provincial study review board that would be kept abreast of study progress and any adaptions made to the protocol through the period of the study.
