# Supplementary material for: A cluster randomized controlled trial of extending ART refill intervals to six-monthly for anti-retroviral adherence clubs
Source: BMC Infect Dis. 2019 Jul 30;19:674. doi: 10.1186/s12879-019-4287-6 (PMC6664572; doi:10.1186/s12879-019-4287-6)
Supplement: Supplementary file 2 — Patient Informed Consent form. Consent form used for study (DOCX 37 kb) [file 12879_2019_4287_MOESM2_ESM.docx]

# Appendix: Patient informed consent

**
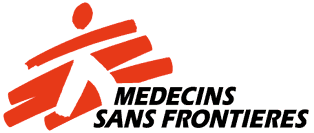
**

***Physical Address:***

***Town One Properties***

***1 Julius Tsolo Street***

***Khayelitsha***

***7784***

***Tel: +27 21 364 5490***

***Fax: +27 21 361 7051***

***Email:*** [***msfocb-khayelitsha@brussels.msf.org***](mailto:msfocb-khayelitsha@brussels.msf.org)

***Web:*** [***www.msf.org.za***](http://www.msf.org.za)

***South African Trust No: IT 577/2011***

**Patient Information Sheet and Informed Consent for participation in the study comparing length of dispensing intervals for ART adherence clubs:**

**5 times a year compared with 6-monthly (twice a year)**

**Principal Investigator**: Lynne Wilkinson, Medecins Sans Frontieres

Proposal Name: **Exploring 6-month dispensing intervals for Adherence Clubs – a cluster randomized study**

This informed consent form has two parts:

- Information Sheet (to provide you with information about the study)
- Informed Consent (for you to provide your consent for participation in the study)

**You will be given a copy of the informed consent form for your record**

**Part I: Information Sheet**

**Introduction**

As a patient receiving your HIV care and ART management including your antiretroviral (ART) drug supply from an ART adherence club at Ubuntu ART clinic, we would like to invite your club to take part in a study about the optimal annual frequency of club visits and length of ART drug supply (i.e. the ideal number of times your club should meet each year which includes the number of months of ART drug supply provided at those visits).

**Purpose**

The purpose of the study is to determine whether patients in clubs which meet:

- twice a year and receive 6 months ART drug supply at each visit; or
- five times a year (every 2 months and 4 month over year end) and receive 2 months ART drug supply each time except at the end of the year when they receive 4 months ART drug supply (current practice at Ubuntu clinic),

have poorer, the same or better outcomes. We will look at whether these patients stay in ART care (in or out of the clubs) and whether their viral loads remain undetectable.

We are carrying out this research to give patients like you an opportunity to take part in research which will influence how often patients who are doing well on ART and are in clubs have to attend their club each year and the length of ART drug supply they receive.

**Type of Research Intervention**

This study will be a cluster randomized trial.

This means that clubs enrolling in the study would either be allocated to:

- the intervention arm that meets twice a year and receives 6-months of ART drug supply at each visit; or
- the standard of care arm, which meet 5 times a year and receives the same ART drug supply at each visit as you do now.

Allocation of each club into either the intervention or standard of care arm will be done randomly. This means your club will have an equal chance of being allocated to either arm. No person will have any influence over this allocation. Participating club numbers will be put into a blank envelope in a sealed box and two independent people (one from MSF and one from Ubuntu clinic) will pull out envelopes. Every second envelope will be allocated to the intervention arm until all the envelopes have been removed from the box.

**Period of the study**

This study will continue for a period of 2 years from the date that your club receives their fourth ART drug supply in 2017 which will be made clear by a researcher/s from the study team (‘Study Officer/s’).

**Study Eligibility**

You are only eligible to participate in this study if you are older than 18 years, you started ART more than 6 months ago and your viral load is undetectable.

**Voluntary Study participation**

Your participation in this study is voluntary and you should not feel pressurized by your club or specific members of your club to vote to in favour or against participation. This is your decision and your vote will be kept confidential and remain unknown to the other club members, clinic patients and clinic staff. Your access to HIV management services, ART refills and any other aspect of care, will not be affected by your decision to participate or not to participate.

After the study has been fully explained to your club at the first club meeting for the year, your club can discuss and ask questions. You will then mark on a voting slip whether you are in favour of your club participating in the study or not. The voting slip will have your name on it but will only to be seen by the Study Officers who will keep your vote confidential and will not disclose your vote to other members of your club.

You will place your folded voting slip in a closed box which will only be handled by the Study Officers. The Study Officers will count the votes outside of the club meeting room and come back to report the final result.

If 90% or less of your club members voted to participate in the study, your club will not be enrolled in the study and you will continue to receive your club care in the same way as you have before. Nothing will change.

If more than 90% of your club members voted to participate in the study, the club will be enrolled into the study. Importantly this does not mean that you have to participate in the study if you do not want too.

The Study Officers will then hand out the informed consent form and explain it to the club members. If you want to be in the study with your club, you can sign the form and hand it to a Study Officer or take it home to read through and bring it back signed to submit to the Study Officer/s at the next club meeting (second club meeting of the year).

On the top of the form there is a place that you can indicate that you do not want to participate in the study and would prefer to transfer to a new club that is not participating in the study. The study staff will not disclose your request to transfer to the other members of the club and will manage your transfer for you confidentially. The Study Officers will make sure that you are allocated to a new club that meets at a time and place that is convenient for you.

If you do not have time to listen to the informed consent process and need to leave for any reason, you can take the informed consent form with you, read at home and a Study Officer will make a plan to meet you and go through it and answer any of your questions just before or after your second club meeting for the year or at another time convenient to you. If you lose the form, do not worry, a Study Officer will give you a new one.

Once the study team has completed this process for all clubs over the first two club meetings of the year and determined which clubs are participating in the study, the clubs will be randomly allocated to either the intervention or standard of care arm (as previously explained).

At your third club meeting for the year, the Study Officers will inform your club which arm of the study your club has been allocated too. You will again be given a voting slip. This time you will mark if you want your club to withdraw from the study. The voting will again be confidential, only the Study Officers will know of your vote and will only report the withdrawal outcome to the group.

If 10% or more of your club members voted to withdraw from the study, your club will be withdrawn from the study and you will continue to receive your club care in the same way as you have before. Nothing will change.

If less than 10% of your club members voted to withdraw from the study, the club will remain enrolled in the study and will be informed how much ART supply your club will receive at their next club meeting (the fourth club meeting for the year). Importantly this does not mean that you have to participate in the study if you do not want too, you can withdraw and transfer to a new club that is not participating in the study. The second voting slip will have a box for you to indicate if you want to transfer out of your club if your clubs remains in the study. Again the study staff will not disclose your request to transfer to the other members of the club and will manage your transfer for you confidentially, making sure that you are allocated to a new club that meets at a time and place that is convenient for you.

It is also important to understand that you can withdraw from the study at any point after signing the informed consent form by informing any of the study team, one of whom will always be present before and during your club meetings. The study team will confidentially arrange for your transfer to another club.

**Procedures**

If your club agrees to participate in this study and neither your club nor you withdraw for any reason, you will receive your HIV care and ART supply as follows:

Intervention arm

At the fourth club meeting in 2017, each club member will be supplied with 6-month of ART and provided with a club return date 6 months later. The Study Officers will be present to explain to all club members the importance of:

- adhering to their ART during the following 6 months;
- bringing an appropriate bag to your club meeting that will accommodate your 6 months ART supply so that it cannot be seen by any community members when you return home on foot or by taxi who may take the opportunity to steal it from you if seen;
- keeping your ART drug supply safe and secure in your home environment. This means it is important to keep your ART supply away from the windows, out of direct sunlight and away from direct heat sources (e.g. a stove or gas ring). It is also better to keep it in a container, cupboard or bag so that it is not visible to anyone who enters your home who may want to take the opportunity to steal it from you if seen;
- reporting to a Study Officer if any of your ART supply is stolen, lost, damaged or destroyed for any reason. There will be no negative consequences and you will immediately receive a replacement ART supply;
- coming to Ubuntu clinic immediately (no appointment required) should you at any time feel unwell, be worried about any symptoms or lose more than 5% of your body weight (if not on a diet with this purpose);
- reporting to Ubuntu clinic reception or the Study Officer if you intend to move and transfer your care to a different ART clinic elsewhere in South Africa; and
- contacting or meeting up with fellow club members outside of scheduled club visits to continue to provide more frequent support amongst one another if this is helpful to you or others in your group.
- reporting to the Study Officer if you have any other problem for example feeling unsupported by your club or clinic or not getting access to healthcare at the clinic.

The Study Officer will also inform your club whether your next scheduled club visit is a clinical club visit or not. If it is a clinical club visit, each member of the club will be required to come to the clinic any day of the week from 2-6 weeks before their next club visit date to have their viral load taken. You will be given a blood requisition slip which indicates that you need to have your viral load taken and will report with this slip directly to the specific room for blood taking as indicated by the Study Officer. You will not be required to collect your folder in reception. It is important that you attend the clinic to have your blood taken to ensure that your result is available at your clinical club visit. If you do not come before your clinical club visit, your viral load will be taken on the day of your clinical club visit and you will be required to come back to the clinic 5 days later for your clinical review and to receive your 6-month supply of ART. You will be given enough ART supply to ensure you do not run out before you receive your 6-month supply.

The Study Officer/s will be present before and at each of your club visits for the period of the study to answer any questions, provide any required support and make sure that if your club’s next visit is a clinical club visit you are issued with blood requisition slips and are aware that you need to attend the clinic before your clinical club visit to have your blood drawn.

Standard of care arm

At the fourth club visit in 2017, the Study Officer will be present at your club meeting to explain that your club will receive HIV care and ART drug supply in the same way as you did before participation in the study and to answer any questions.

**Early study termination**

After 1 year of the study, an independent data monitoring committee will determine whether too many patients in the intervention clubs are being lost from care or have high viral loads. If this is the case, they will recommend the study stop early. If the study is stopped early and your club was part of the intervention arm, you will go back receiving HIV are and ART supply in the club as you did before the study (5 times a year). You will also be asked to return any ART supply you have at home which will be replaced with a new supply.

**End of the study**

At the end of the period of the study, if your club was part of the intervention arm, it will return to receiving HIV care and ART supply in the club as you do now (5 times a year) unless the Western Cape Department of Health changes its policy before the end of the study period. The study team will keep your club informed throughout the period of the study of any expected policy change.

**Risks/Discomforts**

Participation in this study will not impact the care you receive. All treatment and clinical care will remain free of charge to you.

Participation in this study involves the disclosure of your HIV status to the study team members; the study team members will not share confidential information with anyone other than study staff.

If your club is allocated to the intervention arm, at the end of the study, the frequency of club visits to the clinic and the length of ART drug supply may return to what it is today – 5 times a year (2 months ART drug supply at each visit except at year end where you receive 4 months ART drug supply).

**Benefits**

There are no direct benefits involved in participation in this study other than reduced transport, child care and loss of working hours due to reduced club visit frequency and increased length of ART drug supply if your club is allocated to the intervention arm for the period of the study. However, your participation will influence how often patients who are doing well on ART and are in clubs have to attend their club each year and the length of ART drug supply they receive.

**Confidentiality**

If you participate in this study, your clinical records including your blood results in your patient clinic folder, your club’s register, from the national laboratory service and the clinic database will be extracted for the purpose of the research.

All such information will be kept confidential. All study data will be entered into password protected databases to which only study staff will have access. Identifying information will be removed for analytic purposes. All paper study documents (including voting slips and the informed consent forms) will be kept in a locked cabinet at the MSF office and will destroyed 3 years after the final study report has been completed by a company which destroys confidential document.

The results of the study will be collated and the collective findings may be published in future, however all personal information, club attendance and viral load results will remain anonymous.

**Right to Refuse or Withdrawal**

As previously stated your participation is voluntary. If you provide consent to participate and at any point decide your no longer wish to take part in the study you may withdraw your written consent by informing the Study Officer or any of the people listed below.

**Who to Contact**

If you would like any additional information regarding this study please feel free to contact:

*UCT Research Ethics Committee*

Telephone: +27 (21) 406 6492

*Lynne Wilkinson, MSF Principal Investigator*

Telephone: XXXXXXXXXXXXX

*XXXXXXXXXXXX, MSF HIV Epidemiologist, Co-Investigator*

Telephone: XXXXXXXXXXXXX

*XXXXXXXXXXXx, MSF Khayelitsha Co-ordinator*

Telephone: XXXXXXXXXXXx

*XXXXXXXX Ubuntu ART Facility Manager*

Telephone: XXXXXXXXXXXXXX

**This proposal has been reviewed and approved by the Human Research Ethics Committee of University of Cape Town, which is a committee who are responsible for ensuring research participants are protected from harm.**

**Name of club member ………………………………………..**

**Club number ……………………………………………………**

**Patient clinicom number …………………………………………………**

**Indicate by ticking this box if you do not want to participate in the study and would like to transfer to a club that is not taking part in the study□**

***Please insert your current telephone number so that the Study Officer can contact you to arrange your transfer to another club*: ­­­­­­­­­­­­­­­……………………………………….**

**Part II: Informed Consent**

I have read and understood the information provided to me in the information sheet. If I am unable to read someone has read it to me and I understand the information provided. I was given the opportunity to ask questions regarding the study and the study staff provided me with comprehensive answers. I provide my voluntary, written consent to participate in this study and understand that I can withdraw my consent to take part at any point. I understand that if I decide not to take part in this study it will not affect my HIV care or ART drug supply in any way.

Written Consent: _____Yes _____No

Print Name of Participant ______________________

Signature of Participant _______________________

Date (DD/MM/YYYY) _________________________

**If Illiterate**

Thumb print of participant:

Print Name of Study Staff Taking Consent _______________________

Signature of Study Staff Taking Consent ________________________

Date (DD/MM/YYYY) _______________________________________

**A copy of this informed consent form has been given to the patient.**
