# Supplementary material for: A cluster randomized controlled trial of extending ART refill intervals to six-monthly for anti-retroviral adherence clubs
Source: BMC Infect Dis. 2019 Jul 30;19:674. doi: 10.1186/s12879-019-4287-6 (PMC6664572; doi:10.1186/s12879-019-4287-6)
Supplement: Supplementary file 3 — Randomization SOP. The Standard operating Procedure for how randomization was performed during study. (DOC 132 kb) [file 12879_2019_4287_MOESM3_ESM.doc]

# Randomization SOP

The epidemiologist (TC) who is not involved in recruitment, treatment allocation, and outcome assessment will generate the random allocations. Randomization will take place after the consent process is complete and the number of participating community and facility clubs is known. Using the Randomize package in Stata, clubs will be assigned to either the intervention or control arm, while ensuring balance between club type in each arm. Stata code is provided in appendix 1 and an example output is shown in appendix 2.

The epidemiologist will put the assignment (“6-month” or “2-month”) into sealed, opaque, and sequentially numbered, otherwise identical, envelopes. A sheet of carbon paper will also be placed on top of the assignment. The envelopes will be stored in order in two separate boxes, one for community clubs and one for facility clubs. The boxes will be stored in a secure cabinet and the relevant box will be brought by study staff to club meetings.

At the club visit the study supervisor or nurse will take an envelope sequentially from the relevant box, write the club number on the envelope. This process will also ensure that the club number is documented, via the carbon paper, onto the assigned group, before the envelope is opened. A witness will sign next to the club name to verify that the envelope was sealed before opening. The envelope will then be opened and club members will be informed of their assignment and continue with the study procedures. This process is illustrated in appendix 3.

## Appendix 1: Stata Code

ssc install randomize

clear

****number of clubs =90

set obs 90

gen number =_n

***number of community clubs = 31

gen community=1 if number<=31

replace community=0 if number>31

randomize, balance(community) minruns(1000)

label define ass 1 "2-month supply" 2 "6-month supply"

label values _ass ass

***checks

tab _ass

tab _ass comm

## Appendix 2: Example Randomization list


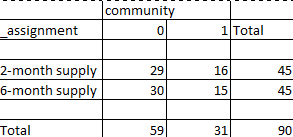


| number | community | _assignment |
| --- | --- | --- |
| 1 | 1 | 2-month supply |
| 2 | 1 | 2-month supply |
| 3 | 1 | 6-month supply |
| 4 | 1 | 6-month supply |
| 5 | 1 | 2-month supply |
| 6 | 1 | 6-month supply |
| 7 | 1 | 2-month supply |
| 8 | 1 | 2-month supply |
| 9 | 1 | 2-month supply |
| 10 | 1 | 2-month supply |
| 11 | 1 | 6-month supply |
| 12 | 1 | 2-month supply |
| 13 | 1 | 2-month supply |
| 14 | 1 | 6-month supply |
| 15 | 1 | 6-month supply |
| 16 | 1 | 6-month supply |
| 17 | 1 | 2-month supply |
| 18 | 1 | 6-month supply |
| 19 | 1 | 6-month supply |
| 20 | 1 | 2-month supply |
| 21 | 1 | 2-month supply |
| 22 | 1 | 2-month supply |
| 23 | 1 | 2-month supply |
| 24 | 1 | 6-month supply |
| 25 | 1 | 6-month supply |
| 26 | 1 | 2-month supply |
| 27 | 1 | 6-month supply |
| 28 | 1 | 2-month supply |
| 29 | 1 | 6-month supply |
| 30 | 1 | 6-month supply |
| 31 | 1 | 6-month supply |
| 32 | 0 | 2-month supply |
| 33 | 0 | 2-month supply |
| 34 | 0 | 6-month supply |
| 35 | 0 | 2-month supply |
| 36 | 0 | 2-month supply |
| 37 | 0 | 2-month supply |
| 38 | 0 | 6-month supply |
| 39 | 0 | 2-month supply |
| 40 | 0 | 6-month supply |
| 41 | 0 | 6-month supply |
| 42 | 0 | 6-month supply |
| 43 | 0 | 2-month supply |
| 44 | 0 | 6-month supply |
| 45 | 0 | 2-month supply |
| 46 | 0 | 2-month supply |
| 47 | 0 | 2-month supply |
| 48 | 0 | 6-month supply |
| 49 | 0 | 6-month supply |
| 50 | 0 | 6-month supply |
| 51 | 0 | 6-month supply |
| 52 | 0 | 6-month supply |
| 53 | 0 | 6-month supply |
| 54 | 0 | 6-month supply |
| 55 | 0 | 2-month supply |
| 56 | 0 | 6-month supply |
| 57 | 0 | 2-month supply |
| 58 | 0 | 2-month supply |
| 59 | 0 | 6-month supply |
| 60 | 0 | 2-month supply |
| 61 | 0 | 6-month supply |
| 62 | 0 | 6-month supply |
| 63 | 0 | 2-month supply |
| 64 | 0 | 2-month supply |
| 65 | 0 | 6-month supply |
| 66 | 0 | 6-month supply |
| 67 | 0 | 6-month supply |
| 68 | 0 | 6-month supply |
| 69 | 0 | 6-month supply |
| 70 | 0 | 6-month supply |
| 71 | 0 | 2-month supply |
| 72 | 0 | 6-month supply |
| 73 | 0 | 2-month supply |
| 74 | 0 | 2-month supply |
| 75 | 0 | 2-month supply |
| 76 | 0 | 2-month supply |
| 77 | 0 | 2-month supply |
| 78 | 0 | 2-month supply |
| 79 | 0 | 6-month supply |
| 80 | 0 | 2-month supply |
| 81 | 0 | 2-month supply |
| 82 | 0 | 6-month supply |
| 83 | 0 | 2-month supply |
| 84 | 0 | 6-month supply |
| 85 | 0 | 6-month supply |
| 86 | 0 | 2-month supply |
| 87 | 0 | 2-month supply |
| 88 | 0 | 2-month supply |
| 89 | 0 | 6-month supply |
| 90 | 0 | 6-month supply |

## Appendix 3: Example Process

| number | community | _assignment |
| --- | --- | --- |
| 1 | 1 | 2-month supply |


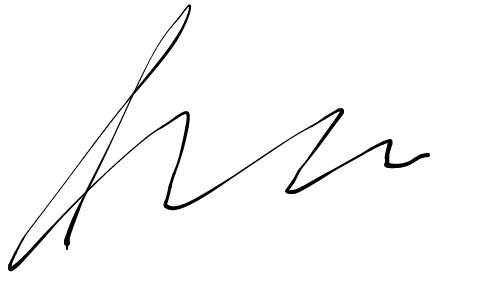

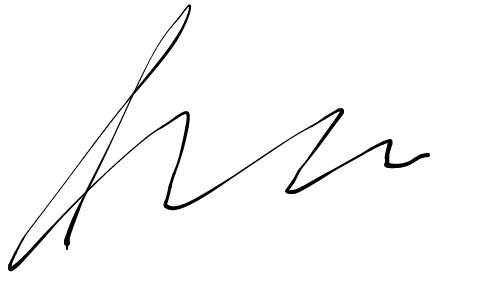


Drawn from box at club meeting and club name and signature is written on envelope

2-month supply

#1

(carbon paper)

**+**

#1

Sealed & placed in box

#1

*Community club 32*

Opened and revealed

Epidemiologist

Study staff

2-month supply

*Community club 32*
